# CRISPR/Cas9-mediated mosaic mutation of *SRY* gene induces hermaphroditism in rabbits

**DOI:** 10.1042/BSR20171490

**Published:** 2018-03-21

**Authors:** Yuning Song, Yuanyuan Xu, Mingming Liang, Yuxin Zhang, Mao Chen, Jichao Deng, Zhanjun Li

**Affiliations:** Jilin Provincial Key Laboratory of Animal Embryo Engineering, Jilin University, Changchun 130062, China

**Keywords:** CRISPR/Cas9, chimerism, hermaphrodite, rabbit

## Abstract

Hermaphroditism is a rare disorder that affects sexual development, resulting in individuals with both male and female sexual organs. Hermaphroditism is caused by anomalies in genes regulating sex determination, gonad development, or expression of hormones and their receptors during embryonic development during sexual differentiation. *SRY* is a sex-determination gene on the Y chromosome that is responsible for initiating male sex determination in mammals. In this study, we introduced CRISPR/Cas9-mediated mutations in the high-mobility-group (HMG) region of the rabbit *SRY*. As expected, *SRY*-mutant chimeric rabbits were diagnosed with hermaphroditism, characterized by possessing ovotestis, testis, ovary and uterus simultaneously. Histopathology analysis revealed that the testicular tissue was immature and lacked spermatogenic cells, while the ovarian portion appeared normal and displayed follicles at different stages. This is the first report of a rabbit hermaphroditism model generated by the CRISPR/Cas9 system. This novel rabbit model could advance our understanding of the pathogenesis of hermaphroditism, and identify novel therapies for human clinical treatment of hermaphroditism.

## Introduction

Hermaphroditism is a rare sex development disorder that affects 0.018% of the human population. Hermaphroditism is caused by anomalies in sex differentiation, gonad development, sex hormones and receptors expression patterns during embryonic development, and result in inconsistencies between the genetic and phenotypic sex [3,4]. Hermaphrodites possess both ovarian and testicular tissues, either in two separate gonads or in one gonad (ovotestis) [7]. Unfortunately, the gender of hermaphrodites cannot be distinguished accurately at birth, causing severe negative consequences to the patient’s physical, mental and psychological status.

*SRY* is a sex-determination gene on the Y chromosome of mammals that is responsible for the initiating male sex determination [8]. Mutations in the high-mobility-group (HMG) box, a conserved DNA-binding domain of SRY, induces hermaphroditism syndrome in XY individuals [12]. There is no consensus on how to treat patients with hermaphroditism, however, surgical interventions are often mandatory [3,6]. Our understanding of sex development disorders remains rudimentary and animal models for clinical research of hermaphroditism syndromes are needed to advance research on genetic hermaphroditism.

CRISPR/Cas9 system uses Cas9 nuclease from bacteria and a single guide RNA (sgRNA) that targets the Cas9 to a gene of interest, and has been applied to edit genomes of various animals [2,5,18,21]. Using CRISPR/Cas9, F0 animals often are genetic mosaics [24], and the mosaic individuals can be important for studying genes that are required for early development by studying the mosaic surviving individuals for the role of the gene of interest in different tissues and cell types. In addition, CRISPR/Cas9 can generate results in one generation, instead of the two or more generations required for using the Cre/LoxP system [10,15]. Here, we generated rabbit carrying mutations in the *SRY* gene by generating CRISPR/Cas9-mediated chimera mutations, and found that the SRY-mutant chimeric rabbits displayed typical phenotypes of hermaphroditism syndromes.

## Materials and methods

### Ethics statement

The rabbits used in this study were New Zealand white rabbits that were obtained from the Laboratory Animal Center of Jilin University. All animal studies were conducted according to the experimental practices and standards approved by the Animal Welfare and Research Ethics Committee of Jilin University.

### Vector construct and *in vitro* transcription

sgRNAs were designed using http://crispr.mit.edu/. Two complementary DNA oligonucleotides were annealed at 95°C for 5 min, then cooled down to 25°C to generate the double-strand DNA (dsDNA) fragments. The pUC57-Simple vector (Addgene ID 51306) was digested with *BbsI* and gel purified using a gel extraction kit (Tiangen, Beijing, China), and the dsDNA for the target sgRNA sequence was cloned into the BbsI-linearized pUC57-T7 vector (Addgene ID 51306). The sgRNAs oligonucleotides sequences targeting *SRY* were listed in Supplementary Table S1. The recombinant vector (Puc57-T7-sgRNA) was amplified with T7 primers (T7-F: 5′-GAAATTAATACGACTCACTATA-3′ and T7-R: 5′-AAAAAAAGCAC CGACTCGGTGCCAC-3′). The PCR products of sgRNA were transcribed using the MAXIscript T7 Kit (Ambion) and purified by with miRNeasy Mini Kit (Qiagen) following the manufacturer’s instructions.

The Cas9 *in vitro* transcript vector, 3xFLAG-NLS-SpCas9-NLS, was synthesized and cloned into the pCS2+vector (Addgene ID 51307). The recombinant Cas9 expression vector was linearized with *Not* and transcribed *in vitro* with the mMessage mMachine SP6 Kit (Ambion, U.S.A.) and the RNeasy Mini Kit (Qiagen) following the manufacturer’s instructions. Cas9 and guide RNA (gRNA) mRNAs quality and concentrations were measured using Nandrop 2000 and agarose gel (1.5%) electrophoresis, respectively.

### Microinjection and embryo transfer

We previously described a protocol for microinjection of pronuclear-stage embryos [18]. In brief, rabbit zygotes were collected from the sexually mature female New Zealand white rabbits (6–8 months old), which were superovulated with follicle-stimulating hormone (FSH) for six times at a 12 h interval. Then, these female rabbits were mated with the male rabbits that were intravenously injected with 100 IU human chorionic gonadotrophin (hCG). 18 h after the hCG injection, the female rabbits were killed, and the oviducts were then flushed with 5 ml DPBS-BSA to collect embryos. Rabbit embryos at the pronuclear stage (around 18–20 h post-mated) were collected and transferred into oocyte manipulation medium, which consisted of 9.5 g TCM-199, 0.05 g NaHCO_3_ (Sigma, S4019), 0.750 g Hepes (Sigma, H3784), 0.05 g penicillin, 0.06 g streptomycin, and 1.755 g NaCl, 3.0 g BSA in 1 l Milli Q H_2_O.

A mixture of *in vitro*-transcribed sgRNAs (25 ng/μl) and Cas9 (100 ng/ul) was injected into the cytoplasm of embryos. The injected embryos were transferred to EBSS medium for 30–60 min, and were then transferred into the oviducts of recipient mothers, where approximately 30–50 embryos were transferred per rabbit.

### PCR detection of mutations in blastocysts and pups

Each injected zygote was collected at the blastocyst stage and incubated in embryo lysis buffer at 50°C for 20 min and 90°C for 5 min. Genomic DNA from *SRY* knockout (KO) rabbit pups was isolated using the TIANamp Genomic DNA Kit (TIANGEN, Beijing, China) following the manufacturer’s instructions. DNA was amplified with 2× Taq Plus MasterMix (TIANGEN). PCR primers used to detect mutation are listed in Supplementary Table S1. PCR products were gel purified and cloned into a pGM-T vector (Tiangen, Beijing, China), and ten positive plasmid clones were sequenced then analysed using DNAman.

### T7 endonuclease I assay

T7 endonuclease I (T7E1) endonuclease I assays were performed as previously described [18]. Briefly, genomic DNA of blastocysts and newborn pups were amplified by PCR with primers listed in Supplementary Table S1. The PCR products were digested with T7E1 (NEB M0302L) and analysed on a gel.

### The off-target assay

Five potential off-target sites (POTS) for each sgRNA were predicted by an online design tool (http://crispr.mit.edu/). To test for off-target effects, PCR products of the POTS were sequenced and confirmed by T7E1 enzyme digestion. Primers for POTS determination were listed in Supplementary Table S2.

### Histology analysis

Hematoxylin and Eosin (H&E) staining was performed following published protocols [25]. Reproductive tissues, including the ovotestis, testis, ovary, fallopian tube, uterus and cervix from *SRY* KO rabbits were fixed with 4% paraformaldehyde for 48 h, embedded in paraffin wax, cut into 5 μm sectioned and then stained with H&E and analysed by microscope (Nikon ts100).

## Results and discussion

**Figure 1:**
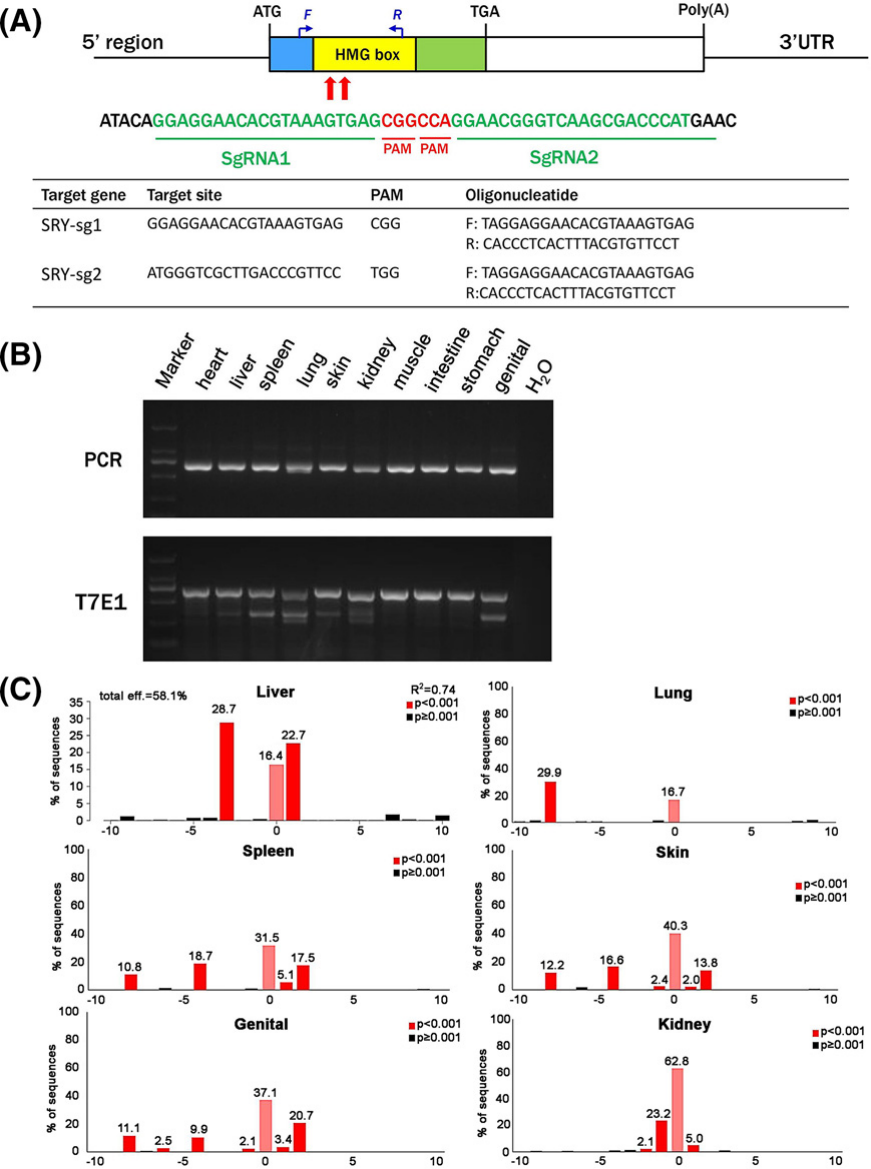
CRISPR/Cas9-mediated editing of *SRY* in rabbits (**A**) Schematic diagram of the two sgRNAs targeting the *SRY* loci. The yellow rectangle represents the HMG region of *SRY*. Two sgRNAs sequences, sgRNA1 and sgRNA2, are highlighted in green. Protospacer adjacent motif (PAM) sequences are in red. Primers F and R were used to detect mutations in the blastocysts and pups. (**B**) PCR and T7E1 cleavage assay for detecting mutations in organs of the *SRY*-mutant chimeric rabbits. M, D2000. (**C**) TIDE mutation detection assay for multiple organs in the SRY-mutant chimeric rabbits. Deletions are denoted as ‘−’ and numbers represent the percentage of the mutant type.

To generate *SRY*-mutant chimeric rabbits, we designed two gRNAs to target the HMG region of the *SRY* gene, which is the DNA binding domain that plays an important role in embryonic development and sex differentiation (Figure 1A). We selected two sgRNAs that were predicted to be specific for *SRY*’s HMG region using CRISPR Design (http://crispr.mit.edu/).

To determine the efficiency of the CRISPR/Cas9 system in editing *SRY, in vitro* transcribed Cas9 mRNA (100 ng/μl) and sgRNA (25 ng/μl) were microinjected into zygotes and cultured until the blastocyst stage. 13 of 30 injected embryos were male and developed to the blastocyst stage; PCR products from these 13 blastocysts were sequenced to determine CRISPR/Cas9’s efficiency in generating mutations in *SRY* in zygotes. Twelve of thirteen of the blastocysts carried a mutation in *SRY* and the mutation efficiency was 92.3% in male blastocysts. Together, this suggested that dual sgRNA directed CRISPR/Cas9 was effective in generating mutations in rabbit *SRY* (Supplementary Figure S1).

We then injected 75 zygotes with a mixture of *in vitro*-transcribed Cas9 mRNA (100 ng/μl) and sgRNA (25 ng/μl) and implanted blastocysts into two pseudo-pregnant recipient females. One of the recipients carried the pregnancies to term and gave birth to a live pup. To examine the whole-body chimeric rate in the founder rabbit, we extracted genomic DNA from the heart, liver, spleen, lung, skin, kidney, muscle, intestine, stomach and genital and tested for mutations in *SRY*. We conducted a T7E1 digestion assay and found that the founder rabbit was mosaic for *SRY* mutations (Figure 1B). We also used an online tool, TIDE [1], to determine editing rates of the target locus in our chimeric rabbit (Figure 1C). The results were confirmed by TA cloning and sequencing, and we found that the founder rabbit carried multiple mutant genotypes, ranging from 1 to 11 bp deletions, in most tested organs (including liver, spleen, lung, skin, kidney and genital) (Supplementary Figure S2). Taken together, we successfully generated SRY-mutant chimeric rabbits using CRISPR/ Cas9.

One of the major concerns of CRISPR/Cas9 is off-target mutations, which has been widely reported in human cell lines, mice [22] and zebrafish [19]. To test whether off-target mutations occurred in our *SRY*-mutant chimeric rabbits, we analysed ten potential off-target sites by Sanger sequencing and T7E1 assay. None of the off-target sites contained mutations, suggesting that no off-target effects occurred in the *SRY* mosaic rabbit (Supplementary Figure S3).

**Figure 2:**
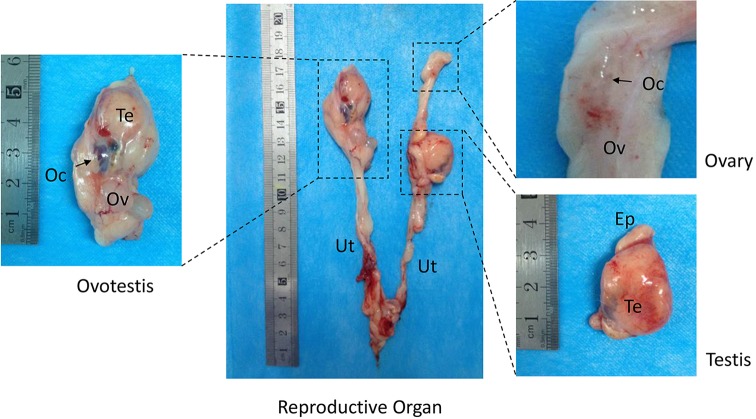
Macroscopic overview of the internal genital structures from *SRY*-mutant chimeric rabbits The gonadal samples consisted of three substantive sections: ovotestis, ovary and testis. Oc, oocytes; Ov, ovary; Te, testis; Ep, epididymis; Ut, uterus.

Next, to determine whether chimeric mutations in the *SRY* gene induces hermaphroditism, we examined the internal genitalia of *SRY*-mutant chimeric rabbits when the pups were 4 months old. The gonadal samples consisted of three substantive sections, including ovotestis, ovary (Ov) and testicular (Te) structure, and there was a wide range of phenotypic ambiguity (Figure 2). The ovotestis contained both testicular and ovarian morphologies.

**Figure 3:**
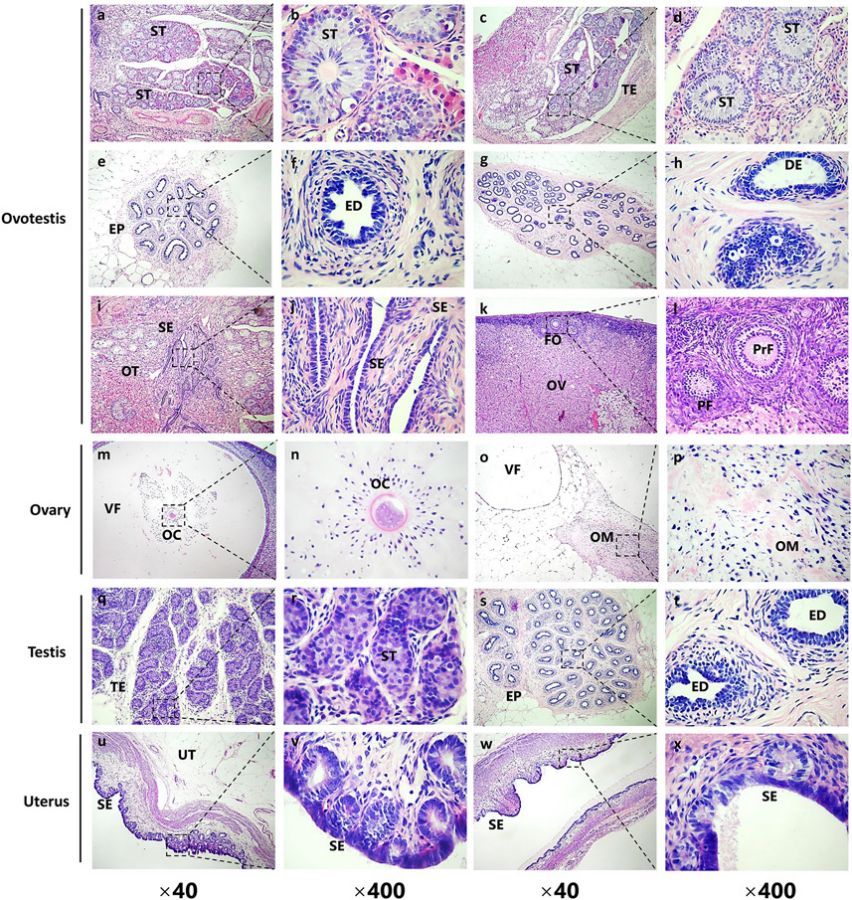
Histological examination of the gonadal structures by H&E staining from *SRY*-mutant chimeric rabbits (**a–l**) H&E staining of the left gonad (ovotestis in Figure 2) from the *SRY*-mutant chimeric rabbit. (**a–h**) Male-like gonadal structures of the ovotestis. ST, seminiferous tubules; TE, testis; EP, epididymis; ED, efferent ductules; DE, ductus epididymidis. (**i-l**) Female-like gonadal structures of the ovotestis. SE, surface epithelium of the endometrium; OT, ovotestis; FO, follicles; OV, ovary; PF, primordial follicles; PrF, primary follicles. (**m–p**) H&E staining of the upper right gonad (ovary in Figure 2) from the *SRY*-mutant chimeric rabbit. VF, vesicular follicles; OC, occytes; OM, ovary medulla. (**q–t**) H&E staining of the bottom right gonad (testis in Figure 2) from the *SRY*-mutant chimeric rabbit. ST, seminiferous tubules; TE, testis; EP, epididymis; ED, efferent ductules; (**u–x**) H&E staining of the uterus from the *SRY*-mutant chimeric rabbit. UT, uterus; SE, surface epithelium of the endometrium.

We prepared serial sections of the internal genitalia for histological examination via H&E staining [17]. The cystic gonad on the left uterine horn contained portions of both the male and female gonadal structures (ovotestis, Figure 2), including testicular parenchyma with seminiferous tubules (ST) (Figure 3a–d), efferent ducts (ED) (Figure 3e,f) and incompletely developed epididymis (EP) with ductus epididymidis (DE) (Figure 3g,h). In the ovotestis, there was a compact arrangement of seminiferous tubules lined with sertoli cells. However, we did not observed germ cells in the seminiferous tubules, and instead found large numbers of interstitial cells near the capsule of both gonads. In the structures that were female-like in the ovotestis, the gonads were covered with cuboidal epithelium, which had the characteristic of the ovarian surface epithelium (SE) (Figure 3i,j). Moreover, different stages of ovarian follicles, including primordial follicles (PF) and primary follicles (PrF), were found adjacent to the ovotestis, some even with oocytes (OC) in it (Figure 3k,l).

The gonadal structure on the upper right side was solely composed of ovarian tissue (Figure 2). There were a few well-developed follicles with oocytes found in the ovarian tissue, which were lined with multilayered ciliated columnar epithelial cells (Figure 3m,n). The vesicular follicles (VF) were embedded into the proliferating connective tissue, and we did not observe corpora lutea (Figure 3o,p). The gonadal structures on the bottom right side of the gonads consisted of testis and epididymis (Figure 2), containing irregularly shaped seminiferous tubules lined with sertoli cells and proliferating interstitial cells. However, there were no spermatogenic cells (Figure 3q,r). In addition, different portions of ductal structures were present in the epididymidis. However, in contrast with the ovary, gametes were absent from the testis (Figure 3s,t). Furthermore, the uterus and uterine glands were lined predominantly with stratified squamous epithelium that were mixed with areas of columnar epithelium (Figure 3u–x).

Genetic mosaics and chimeras are classic experimental subjects that have been employed in both *Drosophila* [13] and mouse [14] genetic studies. Editing the genome of embryos using CRISPR/Cas9 often results in genetically mosaic individuals [2,16], including in zebrafish [19], mice [19,22] and Drosophila [13]. Genetic mosaics are common in CRISPR/Cas9 embryonic injections because genome editing occurs following the one-cell embryonic stage, resulting in diverse genetic outcomes [16,23]. CRISPR/Cas9 can also generate complex alleles, such as tagged proteins and conditional alleles, when co-delivered with longer DNA donor sequences [22]. Using mosaic and complex alleles, cell autonomous and non-autonomous roles of genes can be better studied, and this has already shown great promise in developing cancer study models [11].

Patients with hermaphroditism appear to show regular function of endocrine and gametogenic functions in the ovary or the ovotestis [9]. However, the testis or the testicular portion of the ovotestis shows aberrant hormone production and spermatogenesis [20], which is consistent with the phenotypes observed in the *SRY*-mutant chimeric rabbits in our study. In addition, depletion of the spermatogenic cells may lead to hyperthermia due to the pelvic location of the testis or failure of the germ cells to remain in the primitive seminiferous tubules. Our study is the first report about using CRISPR/Cas9-mediated mosaic genome editing of *SRY* that leads to hermaphroditism in rabbits, demonstrating that CRISPR/Cas9-mediated mosaic genome editing is an important addition to the current collection of genetic tools, particularly for rapidly assaying phenotypes. Meanwhile, this model can be important in understanding the pathogenesis of hermaphroditism and finding novel therapies for the human clinical treatment.

## Supporting information

**Figure S1.** Dual sgRNA-directed mutation of SRY in zygotes. (A) Mutation detection in blastocyst by PCR. M, marker; 1#–13# represent different male blastocysts used in this study. M DNA marker. (B) Mutation detection in blastocyst by T-cloning and Sanger sequencing. The WT sequence is shown at the top of the targeting sequence. sgRNA sequences are marked in green and the protospacer adjacent moti (PAM) sequences are in red with underline. WT: wild type; deletions ‘-’; insertion ‘+’.

**Figure S2.** T-cloning and Sanger sequencing for mutation detection of multiple organs in the SRY-mutant chimeric rabbits. The PAM sites are underlined and highlighted in red; the target sequences are shown in green; the insert sequences are shown in yellow. Deletions (−) and insertions (+) are shown. WT, wild-type control.

**Figure S3.** Off-target analysis of the 10 potential off-target sites (POTS) for sgRNA1 and sgRNA2 in the *SRY* KO rabbits.

**Table S1:** Primers used for construction of sgRNA expression plasmids and PCR mutation detection.

**Table S2:** S2.10 potential off-target sites examined by PCR and primers used for list.

## Abbreviations

gRNA: guide RNA
H&E: Hematoxylin and Eosin
hCG: human chorionic gonadotrophin
HMG: high-mobility-group
KO: knockout
POTS: potential off-target sites
sgRNA: single guide RNA
T7E1: T7 endonuclease I
