# Supplementary material for: CRISPR/Cas9-mediated mosaic mutation of SRY gene induces hermaphroditism in rabbits
Source: Biosci Rep. 2018 Mar 21;38(2):BSR20171490. doi: 10.1042/BSR20171490 (PMC5861328; doi:10.1042/BSR20171490)
Supplement: Supplementary file 1 [file bsr20171490_Supp1.pdf]

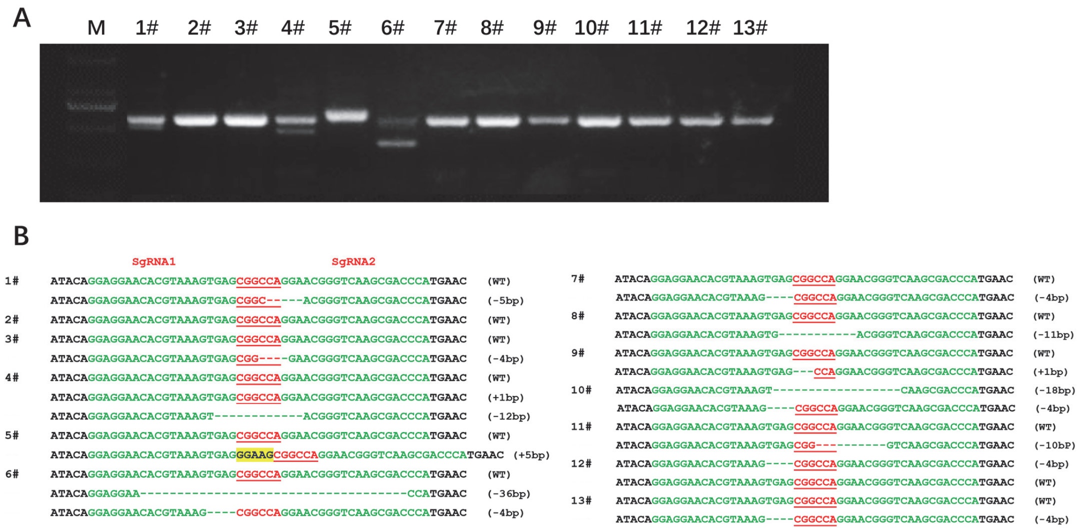

Figure S1. Dual sgRNA-directed mutation of *SRY* in zygotes. (A) Mutation detection in blastocyst by PCR. M, marker; 1#–13# represent different male blastocysts used in this study. M DNA marker. (B) Mutation detection in blastocyst by T-cloning and Sanger sequencing. The WT sequence is shown at the top of the targeting sequence. sgRNA sequences are marked in green and the protospacer adjacent moti (PAM) sequences are in red with underline. WT: wild type; deletions "–"; insertion "+".

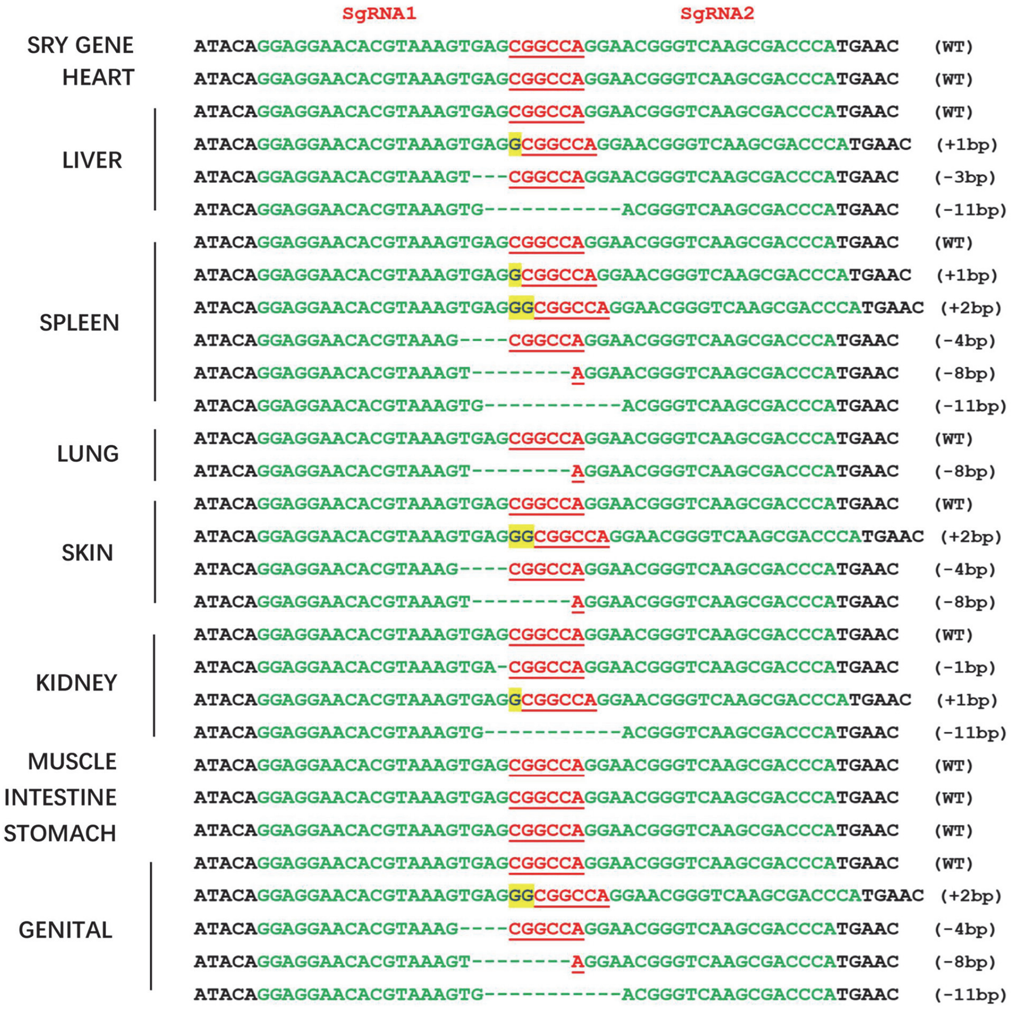

Figure S2. T-cloning and Sanger sequencing for mutation detection of multiple organs in the *SRY*-mutant chimeric rabbits. The PAM sites are underlined and highlighted in red; the target sequences are shown in green; the insert sequences are shown in yellow. Deletions (−) and insertions (+) are shown. WT, wild-type control.

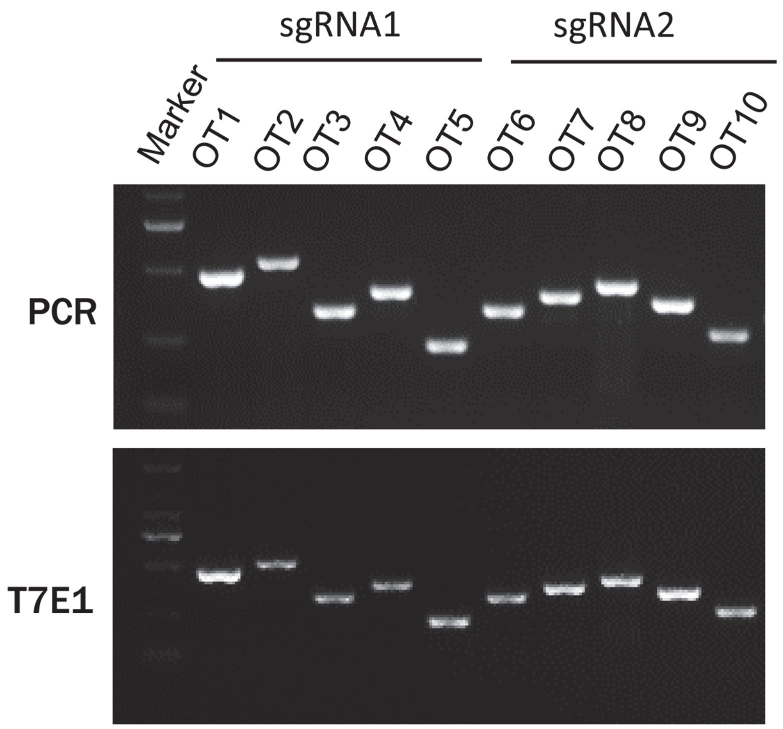

Figure S3. Off-target analysis of the 10 potential off-target sites (POTS) for sgRNA1 and sgRNA2 in the *SRY* KO rabbits.

Table S1: Primers used for construction of sgRNA expression plasmids and PCR mutation detection.

NO.	Name	Primers	Sequence(5'-3')
**1**	sgRNA1	gRNA -1-F	TAGGAGGAACACGTAAAGTGAG
		gRNA -1-R	CACCCTCACTTTACGTGTTCCT
**2**	sgRNA2	gRNA -2-F	TAGGAGGAACACGTAAAGTGAG
		gRNA -2-R	CACCCTCACTTTACGTGTTCCT
**3**	SRY	SRY-F	AGGCATGGAATCCACAATGA
		SRY-R	AATGGGTAGTTTACAGGGAGATG

1 and 2 were used for the construction of sgRNA expression plasmids; 3 was used for the PCR mutation detection.

Table S2.10 potential off-target sites examined by PCR and primers used for list.

	sgRNA Sequence	Potential Off Target Sites	Number of mismatch	Position
s1	GGAGGAACACGTAAAGTGAG	GGAGGAGGACGTAAAGTGAG	2	chr16:-21600211
		GGAGGAACAAGTAAAGTGGG	2	chrUN0:+2958105
		GAAGGAGCAGGTAAAGTGAG	3	chrUN0:-2242557
		ACAGGAATAAGTAAAGTGAG	4	chr12:+15386370
		TGTGGAAAATGTAAAGTGAG	4	chr16:+67886992
s2	TGGGTCGCTTGACCCGTTCC	TGGGCCGCTTGACCCGGTCC	2	chrUN0:+23582
		TGGGGCTCTTGGCCCGTTCC	4	chr15:-4547229
		TGGGTGGCTGGAGCCGTTCC	3	chrUN0:-157366
		TGTGTGCCTGGACCCGTTCC	4	chr5:-1695177
		TGGGCTGCTAGTCCCGTTCC	4	chr1:-194836712
